# If I Were a Doctor

**Published:** 1872-10

**Authors:** Ben. H. Pratt


### THE BISTOURY.

VOL. VIII. ELMIRA, N. Y., OCTOBER, 1872. NO. 3.

### Communications.

### IF I WERE A DOCTOR.

BEN. H. PRATT, A. M.

Not that I wish to be a Doctor, nor that I would prove a better or worse Doctor than the average. In fact, having stood upon the very brink of Medicine, once upon a time, I suppose I might have leaped in, but for reasons not interesting to the reader.

While thus standing and gazing into the vortex of Physic, preparing for the plunge—provided I should decide to plunge—naturally I had laid out my course of conduct toward that very vague and indefinite folk, my prospective Patients. I don't hold that it was presumptuous on my part to people my future world with patients, thousands of them. Yet many Doctors don't find all the patients their youthful fancies conjured up. However, I had them—in imagination—and I'm going to tell you just what I was going to do when they sought for advice and treatment.

Imprimis:—There are but three classes of patients: the Sick, the Ailing, the Hypochondriacal.

Everybody knows when a man is sick. There's no fooling about it, no mistaking it, no dodging it. You know he's in for a bad time—has everybody's sympathy—isn't worth a cent to anybody but the Doctor—down on his back—helpless. You look at him and say "poor fellow," and thank goodness you're on your legs yet. That man's *sick*.

Everybody *don't* know when a case is Ailing. The man is about his business—looks well enough, may be—walks erect, perhaps—eats like other folks—don't get any sympathy from people in general—tries to convey the impression that he's pretty well—gets mad if you hint that he's not—nurses his malady in secret or in private confabs with the Doctor—takes pills, syrups, bitters, powders, boluses, essences, teas—applies blisters,plasters, poultices—rubs, pounds, anoints, bathes--tries everything anybody suggests, every newspaper prescription, every advertised nostrum, in the hope of some day finding himself a well man. He's *ailing*.

But the Hypochondriac is a poser. He's misery. Death looks jolly alongside of him. Seven millions of devils with red hot needles, pricking rattlesnake virus into every pore of his body, would be a delightful solace to him compared with the torture he agonizes under, if you believe all he says of his sufferings. Truly hypochondria is hell, and hell is hypochondria.

These are the three grand divisions into which every Doctor's patients, are divided. There are degrees of each, but these will suffice for our present purpose.

The Doctor *goes* to the sick. Sick people don't *come*. Sick people don't do anything, nor say anything; they're sick. The Doctor who relies much upon a sick man's story has a very frail staff to lean upon. The diagnosis is everything. The Doctor becomes at once a judge, and his jury are the pulsations, the tongue, the skin, the temperament. Symptoms are important witnesses, while the statements of the sufferer describing his feelings are but circumstantial evidence. Oft-times the case is clear, from its peculiar nature. An operation becomes necessary at once, perhaps, or the treatment can admit of but one course. Then do quickly, yet deliberately and as well as you know, that which must be done, regardless of personal feelings or any minor considerations. Prompt action, though counter to the wishes of the patient and friends, has saved many a life. Act as though you were sole master of the sufferer's destiny and let nothing warp your course a hair's breadth. Success alone determines what your after position shall be—succeed and be somebody—fail and be nobody; that's a Doctor's daily risk.

It is often, however, a matter of great doubt as to the remedies to be used. Here haste is unpardonable. Careful study, intense thought, close examination, dilligent inquiry into all the circumstances of temperament, habits and character of the patient cannot be too strongly urged. Your diagnosis is then your foundation—build upon it as one whose faith in his substructure is absolute. Then, if fail we must as fail we may, a consciousness of having done the best possible, is the surest ladder out of the pit failure.

In these serious and acute cases, however, there is little discrepancy in practice, the wide differences in treatment occurring in the second and third general classes into which I have divided patients.

The patient who ails, is a difficult subject to treat. He is so sensitive regarding his ailment; so unwilling to admit another into his confidence; so slow to believe that he is really in need of medical aid, that he becomes a self-doctor. He reads up his case—compares his own with known similar cases—tries so many compounds upon himself that he finally concludes his case a desperate one, beyond the reach of human aid. If at last he find it necessary to consult a physician, he does so against his will, under protest, with no confidence in medical help and with no faith in his recovery. The ailing man meets you armed at every point to baffle your skill. He deems you an enemy whom he approaches as a last resort, simply to have his sentence of hopeless invalidism pronounced upon him. How will you meet him? for perhaps it is the first impression you produce upon him which will determine whether or no you are to save him. It is a delicate matter, meeting with this man. He is a stranger perhaps, with peculiarities you may not know or see. Therefore make it no serious matter for him, but with as much pleasantry as consistent with your office, draw from him casually the details of his malady, and you will find in nine out of ten cases that he is a slave and victim to habits, the deleterious effects of which he never dreamed. But you may not tell him this, but so shape your advice as to preclude the indulgence of accustomed habits, and institute for him a course of life the importance of which he must be made to see. Inculcate in his mind faith in yourself—prove yourself more friend than doctor—allude as little to his ailments as possible—get his mind off from himself and fix it upon any object you find convenient. If you can ascertain the bent of his mind—what, aside from himself, he takes most interest in—cultivate that idea by every encouragement possible. Bring to bear all the ridicule you are master of, upon the habits in others, which you are confident he indulges in. Detail warmly but not warningly, the effects of improper courses in others, especially those to which you suspect he inclines. Do it incidentally, concealing your object shrewdly. Such cases need little or no medicine. Simple remedies, and such as have little or no effect save to satisfy the patient that he is "taking" something, are all that are necessary. Bread pills and colored spring-water have accomplished miracles, and made the reputation of many a Doctor. I sometimes feel inclined to doubt the wisdom of withholding instinct from man, and giving him reason in its stead. The brute instinctively avoids eating that which hurts him—drinking that which maddens him—doing that which injures him. Not so with man. He is slow to see the bad effects of anything he enjoys. He suffers again and again for his sins of omission and commission, and learns no lesson. He sees law written in matter—observes cause and effect—scans the connection between the physical and mental—but never applies to himself the wisdom he gains. It's human nature to do so, and the Doctor is wise who accepts the situation, and don't give his patients credit for too much common sense. The Doctor is as bad as the rest in treating his own case, but can do wonders with another, providing he has first impressed that other with his wish and ability to aid and cure.

Then, too, it's a fine point to determine just how far one should make light of the ailings of such a patient, and how far one can dwell seriously upon them. Either course, carried beyond propriety, kicks the work all over. The man is n't a fool, and can't be persuaded that he's not an invalid; and to deal with him as a really sick man, is to drive him from you into his former state. The patient often needs more study than the case, and he is wise who don't do much with the body until he's pretty familiar with the mind.

The Hypochondriac is just the opposite of the Ailer. He is bound to be sick, and determined that everybody shall know it. He's bad. He don't know what the difficulty is, nor where it is, nor where he got it, nor how it affects him. He goes to the doctor—goes to all the doctors.

When asked to state his case, he begins by damning the doctor whose business it is to find out the difficulty for himself. He wants to know what and where the malady is, worse than the doctor. He 's a dying man—dying by inches—can't last till spring—has made his will—doctors are a set of blockheads. The only friend he has is he who will assent to, and believe implicitly in, all the diseases with which he claims to be afflicted. Here then is our clue. To be of any service to such an one, his doctor must admit the desperate nature of his many afflictions, and the more serious he can depict them, the more favor and greater confidence will he inspire. The man is simply a monomaniac, but there is no asylum provided for such. He annoys but don't injure his friends. To cure him he must first be made sick, really sick. This is not difficult nor dangerons. His mind, which is the only diseased portion of this man, must then be led by easy gradations to appreciate a gradual overcoming of the imaginary disease, until at length, with patience and much forbearance, the doctor may effect a cure which may or may not be permanent. If this extraordinary patient can be led to dread the process of healing which he has borne, worse than the disease which he *thinks* he has suffered from, there is little likelihood of a relapse. Otherwise there is danger, and hence it would be policy for the doctor to lead him through as disagreeable a course of nauseating and painful compounds as is consistent with the permanent health of the patient.

A well authenticated case is on record, of a lady, wife of a nobleman in Lisbon, who otherwise entirely sane, imagined that she had a sack of water in her abdomen, which was so rapidly filling up that it would soon burst and kill her. Having called many physicians, each of whom endeavoring to disprove her whim were peremptorily dismissed, a young fledgling in medicine, but nevertheless a shrewd chap, called upon her with the assurance that he could effect a perfect cure. She gladly assented to his treatment. He took a plate, and having smoked it over the flame of a candle, bade her touch the tip of her tongue to the blackened surface of the plate at precisely three minutes past each hour; then told her that when she had done as he had bidden her a certain number of times, the water in the sack would begin to be absorbed, and if she found it absorbing too rapidly, (which she would know by certain described feelings,) she should send immediately for him, and he would change the treatment. The doctor left and awaited patiently the call which he felt pretty certain would come. Sure enough, the servant soon came in breathless, saying that "Missis' bag was drying up too fast,and he should hurry up." In haste the lady was sought, suffering terribly from the rapid collapsing of the sack. Having previously prepared a vial of rain-water colored with cochineal, he straightway administered one drop every sixty seconds, which soon checked the absorption. By alternately applying plate and drop, her case progressed favorably and a perfect and permanent restoration resulted. The lady being a person of distinction, noised abroad the young doctor's wonderful skill, from the effects of which he soon rose to a high position in the profession, and became a brilliant member of society.

